# Estradiol-to-follicle ratio on human chorionic gonadotropin day is a novel predictor of gestational diabetes mellitus in women receiving fresh embryo transfer

**DOI:** 10.3389/fendo.2024.1465069

**Published:** 2024-10-11

**Authors:** Huijun Chen, Yvonne Liu, Xiangwang Xu, Liang Hu, Sufen Cai, Fei Gong, Ge Lin, Philipp Kalk, Bernhard K. Krämer, Berthold Hocher

**Affiliations:** ^1^ Department of Nephrology, Charite´ Universitätsmedizin Berlin, Berlin, Germany; ^2^ Clinical Research Center for Reproduction and Genetics in Hunan Province, Reproductive and Genetic Hospital of CITIC-Xiangya, Changsha, Hunan, China; ^3^ Fifth Department of Medicine (Nephrology/Endocrinology/Rheumatology/Pneumology), University Medical Centre Mannheim, University of Heidelberg, Mannheim, Germany; ^4^ Institute of Reproductive and Stem Cell Engineering, NHCKey Laboratory of Human Stem Cell and Reproductive Engineering, School of Basic Medical Science, Central South University, Changsha, Hunan, China; ^5^ Diaverum Renal Care Center, Diaverum MVZ Am Neuen Garten Standort Ludwigsfelde, Potsdam, Germany; ^6^ Hunan International Scientific and Technological Cooperation Base of Development and Carcinogenesis, Changsha, China

**Keywords:** estradiol: follicle ratio, GDM, predictor, IVF, pregnancy outcomes

## Abstract

**Aims:**

To assess the predictive value of estradiol (E2) related parameters on the incidence of gestational diabetes mellitus (GDM) in women undergoing fresh embryo transfer.

**Materials and methods:**

A *Post-hoc* analysis of a prospective cohort study.

**Results:**

We identified an optimal E2/follicle (E2/F) ratio threshold of 246.03 pg/ml on the day of human chorionic gonadotropin (hCG) administration. Women with an E2/F ratio exceeding this threshold had significantly lower rates of GDM (12.75% vs. 20.41%, P < 0.001) and ovarian hyperstimulation syndrome (OHSS) (11.75% vs. 15.48%, P = 0.03). Additional E2 parameters were also evaluated: baseline E2, E2 on hCG day, E2 increase, and E2 fold change. Lower GDM rates were observed in women with baseline E2 above 31.50 pg/ml (13.51% vs. 19.42%, P <0.01), E2 on hCG day above 3794.50 pg/ml (12.26% vs. 19.32%, P < 0.001), and E2 increase above 3771.50 pg/ml (12.24% vs. 19.28%, P < 0.001). There were no significant differences in OHSS rates for these additional E2 parameters. After adjusting for confounders, lower E2/F ratio (OR: 1.626, 95% CI: 1.229-2.150, P <0.01), E2 on hCG day (OR: 1.511, 95% CI: 1.133-2.016, P = 0.01), and E2 increase (OR: 1.522, 95% CI: 1.141-2.031, P <0.01) were identified as risk factors for GDM.

**Conclusion:**

This study demonstrates that an E2/F ratio over 246.03 pg/ml is significantly associated with a reduced risk of both GDM and OHSS in women undergoing fresh embryo transfer, highlighting the E2/F ratio as a superior predictive biomarker compared to other E2-related parameters.

## Introduction

Gestational diabetes mellitus (GDM) is currently the most common medical complication of pregnancy, and the prevalence of undiagnosed hyperglycemia and even overt diabetes in young women is increasing (1). The risk factors for GDM include advanced maternal age (2), high body mass index (BMI) (3), smoking (4), dietary habits (5–7), family history of type 2 diabetes mellitus (1), previous history of GDM (1) as well as ethnicity (8). Besides these, emerging data indicate a possible contribution of environmental and psychosocial factors to the risk of developing GDM such as polybrominated diphenyl ethers (9) and perfluoro-octanoic acid (10) exposure as well as maternal depression (11).

GDM impairs the gestational process both in maternal and fetal in long-term and short-term prospects. It increases pregnancy complications and adverse fetal events such as pre-eclampsia, preterm birth, shoulder dystocia or birth injury, and clinical neonatal hypoglycemia in the short term (1, 12). Additionally, it also increases the risk of further maternal and offspring diabetes and offspring overweight or obesity (12, 13).

Interestingly, emerging evidence demonstrates that the *in-vitro* fertilization (IVF) treatment also plays a contributing role in developing GDM. There is a well-designed systematic review and meta-analysis which includes 38 studies and a total of 1, 893, 599 women, showing that the singleton pregnancies achieved through assisted reproductive technology (ART) exhibited an elevated risk of GDM in comparison to spontaneously conceived singleton pregnancies (relative risk (RR) 1.53, 95% confidence interval (CI) 1.39–1.69; I^2^ 78.6%, n = 37, 1,893,599 women) (14). Specifically, such a higher risk of GDM was observed only after fresh but not after frozen embryo transfer. The potential explanation might be due to the known adverse effects of ovarian stimulation on endometrial receptivity (15, 16). The significance of promptly identifying GDM in women undergoing ART is underscored by this discovery. Early detection can pave the way for timely and effective interventions, both before ART procedures and during the early stages of pregnancy. However, the reasons for the heightened risk of GDM after ART treatment are not yet fully understood. Furthermore, the data presented does not allow us to discern whether the observed association is attributable to the presence of infertility itself or the specific ART procedures employed (14, 17).

IVF is a complicated process that includes ovarian stimulation, oocyte retrieval, fertilization, embryo culture as well as embryo transfer, featured by supraphysiological estradiol (E2) level. Theoretically, individual IVF steps could play different roles in further developing pregnancy complications such as GDM. Thus, we tried to focus on the IVF-related factors contributing to GDM. Surprisingly, we found that fresh blastocyst embryo transfer increases the risk of GDM, compared to cleavage embryo transfer in our previous study (18). Hence, our study indicates that the IVF procedure itself could contribute to GDM to some extent.

The special significance of GDM risk in IVF pregnancies lies in the combination of age, pre-existing conditions, and IVF-specific factors, all of which require comprehensive management to mitigate risks for both mother and baby. Identifying IVF-related risk factors for GDM is crucial for preventing GDM development after IVF treatment. Thus, we conducted this study to evaluate whether the supraphysiological E2 level affects GDM incidence in future conception.

## Materials and methods

### Study design

This *post-hoc* analysis of the data from our previous cohort (19), was collected from January 2017 to December 2018. Follow-up assessments have been concluded. The study obtained approval from the Ethics Committee at CITIC-Xiangya’s Reproductive and Genetic Hospital under the approval number LL-SC-2018-014, and written consent was obtained from all participating patients.

### Participants

In this study, we included a cohort of 1593 participants who were pregnant after fresh embryo transfer. The specific inclusion and exclusion criteria were previously detailed in the respective references (19, 20) and are outlined as follows:

Inclusion criteria:

1. 18-39 years old
2. first IVF/intracytoplasmic sperm injection (ICSI) cycle
3. received fresh embryo transfer and became pregnant after the confirmation of ultrasound.

The exclusion criteria were:

1. uterine malformations (uterine septum ≥0.6 cm (identified by hysteroscopy or four-dimensional color Doppler ultrasound), single-horned uterus, double uterus)
2. endometriosis
3. intrauterine adhesion
4. untreated hydrosalpinx
5. uterine myoma (multiple, submucous, or intramural myoma >3 cm)
6. oocyte donation cycles
7. pre-implantation genetic test for aneuploid (PGT-A)
8. Cushing syndrome
9. adult-onset adrenogenital syndrome (AGS)
10. any hypothalamic or pituitary disease leading to infertility.

All the participants received an agonist protocol for ovarian stimulation as described in our previous studies (19, 20). We recorded the follicles from the day women started gonadotropin (Gn) to the day with human chorionic gonadotropin (hCG) trigger. Follicles with a diameter over 12mm under transvaginal ultrasound will be measured and recorded on the hCG day as there is a suggestion that follicles with a minimum diameter of 12 mm exhibit favorable rates of fertilization and cleavage (21). Estradiol/follicle (E2/F) ratio is defined as peak E2 level (E2 on hCG day) divided by follicle number on hCG day.

E2 level was assessed by enzyme-linked immunosorbent assay (ELISA) according to the manufacturer’s instruction. Blood samples was collected in the morning on different days. Serum was separated after the centrifugation and evaluated by the automatic machine.

GDM screening was conducted for all participants using an oral glucose load. Specifically, a 75-g oral glucose tolerance test (OGTT) was performed in accordance with guidelines at approximately 20 weeks of gestation or later (22). Pregnant women maintained a normal diet for three consecutive days and fasted for at least 8 hours before undergoing the OGTT. During the test, they consumed 300 mL of water containing 75 g of glucose within 5 minutes. Venous blood samples were collected before and at 1 and 2 hours after glucose intake, using test tubes containing sodium fluoride. Blood glucose levels were measured using the glucose oxidase method. The standard diagnostic criteria for GDM based on the 75-g OGTT are as follows: a blood glucose level of 5.1 mmol/L (92 mg/dL) or higher before glucose intake, 10.0 mmol/L (180 mg/dL) or higher at 1 hour, and 8.5 mmol/L (153 mg/dL) or higher at 2 hours (22).

Ovarian hyperstimulation syndrome (OHSS) is a complication arising from fertility treatments that use pharmacological ovarian stimulation to increase the number of oocytes and embryos available during ART. The diagnosis of OHSS is made on clinical grounds. The typical patient presents with abdominal distension and discomfort following the trigger injection administered to promote final follicular maturation before oocyte retrieval. The diagnosis of OHSS combines symptoms as well as biochemical investigations which is complicated. The OHSS diagnosis in our participants fully complied with the guidelines published by the Royal College of Obstetricians & Gynecologists (23).

### Statistical analysis

The primary data underwent *post-hoc* analysis using Statistical Package for Social Sciences, version 29.0 (SPSS Inc., Chicago, IL, USA). Receiver Operating Characteristic (ROC) analysis was applied to determine the optimal E2/F ratio, E2 on hCG day, and baseline E2 level. The cut-off value was determined by the Youden Index, which equals sensitivity + specificity - 1 according to the ROC results. When the Youden Index is maximized, the corresponding cut-off value is considered the optimal choice. Graphs were created using either GraphPad Prism 8 (GraphPad Software, San Diego, USA) or SPSS. Homogeneity of variance and data normality were evaluated through the Levene and Kolmogorov-Smirnov tests, respectively. Descriptive statistics presented values as frequency (%) or median (interquartile range, IQR). Categorical variables underwent Chi-square (χ2) testing, while continuous variables were assessed using the Mann-Whitney U test. Additionally, a multivariate regression model was developed, considering factors with a significant p-value in the descriptive statistics. A significance level of p < 0.05 was applied for statistical significance.

## Results

Initially, ROC analysis was employed in our investigation to determine the optimal E2/F ratio and its association with the incidence of GDM. An area under the curve (AUC) of 0.572 and a P-value of <0.001 were observed (**Supplementary Figure S1A**). The cutoff value for the E2/F ratio was identified as 246.03 pg/ml using the maximal Youden Index. Subsequently, participants were stratified into two groups based on this cutoff: E2/F < 246.03 pg/ml (n=691) and E2/F ≥ 246.03 pg/ml (n=902).

**Table 1** presents the baseline characteristics, which exhibit significant differences between the two groups. In the group with a higher E2/F ratio, women display lower values in menstrual cycle duration (30.50 (29.00, 37.50) vs. 30.00 (29.00, 34.00), P<0.001), BMI (21.48 (19.82, 23.44) vs. 21.23 (19.53, 22.89), P=0.01), and waist-to-hip ratio (0.82 (0.79, 0.85) vs. 0.81 (0.78, 0.84), P<0.001). Additionally, this group exhibits a superior ovarian reserve, reflected in a higher antral follicle count (AFC) (26.00 (17.00, 30.00) vs. 23.00 (16.00, 30.00), P<0.001). Regarding baseline sex hormone levels, women with a higher E2/F ratio have elevated follicle-stimulating hormone (FSH) (5.53 (4.68, 6.42) vs. 5.63 (4.84, 6.67), P=0.03) and E2 (32.00 (26.00, 42.00) vs. 35.00 (28.00, 45.00), P<0.001) levels, with no significant differences observed in luteinizing hormone (LH), progesterone, testosterone (T), and prolactin (PRL) levels (**Table 1**). Interestedly, we observed that women with higher E2/F ratio have significantly lower systolic (116.00 (109.00, 123.00) vs. 114.00 (108.00, 120.00), P<0.01) and diastolic blood pressure (76.00 (70.00, 82.00) vs. 75.00 (69.00, 80.00), P=0.01).

**Table 1:** The demographic information of participants.

Table 1	E2/F<246.03pg/ml (n=691)	E2/F≥246.03pg/ml (n=902)	P value
Age (y)	29.00 (27.00, 31.00)	29.00 (27.00, 31.00)	0.88
Menstrual cycle (d)	30.50 (29.00, 37.50)	30.00 (29.00, 34.00)	<0.001
Infertility type			
Primary	55.72 (385/691)	55.10 (497/902)	0.81
Secondary	44.28 (306/691)	44.90 (405/902)	
BMI (kg/m2)	21.48 (19.82, 23.44)	21.23 (19.53, 22.89)	0.01
Waist-to-hip ratio	0.82 (0.79, 0.85)	0.81 (0.78, 0.84)	<0.001
Infertility duration (y)	3.00 (2.00, 5.00)	3.00 (2.00, 4.00)	0.24
AMH (ng/ml)	5.74 (3.64, 9.78)	5.80 (3.74, 8.98)	0.65
AFC	26.00 (17.00, 30.00)	23.00 (16.00, 30.00)	<0.001
Basal FSH (mIU/ml)	5.53 (4.68, 6.42)	5.63 (4.84, 6.67)	0.03
Basal LH (mIU/ml)	3.64 (2.63, 5.27)	3.60 (2.60, 4.85)	0.50
Basal E2 (pg/ml)	32.00 (26.00, 42.00)	35.00 (28.00, 45.00)	<0.001
Basal PRL (ng/ml)	14.65 (10.91, 19.40)	15.42 (11.22, 20.22)	0.07
Basal P (ng/ml)	0.23 (0.17, 0.32)	0.24 (0.18, 0.32)	0.67
Basal T (ng/ml)	0.28 (0.23, 0.37)	0.28 (0.23, 0.35)	0.56
Systolic blood pressure (mmHg)	116.00 (109.00, 123.00)	114.00 (108.00, 120.00)	<0.01
Diastolic blood pressure (mmHg)	76.00 (70.00, 82.00)	75.00 (69.00, 80.00)	0.01
Fasting glucose (mmol/L)	5.23 (4.99, 5.48)	5.12 (4.90, 5.38)	<0.01
LH on hCG day (mIU/ml)	1.48 (1.15, 1.92)	1.59 (1.25, 2.04)	0.001
E2 on hCG day (pg/ml)	2760.00 (2122.00, 3568.00)	4314.00 (3469.50, 5113.75)	<0.001
P on hCG day (ng/ml)	0.54 (0.38, 0.71)	0.63 (0.47, 0.84)	<0.001
Follicles on hCG day	14.00 (11.00, 18.00)	13.00 (11.00, 16.00)	<0.001
The number of embryos transferred			
Day 3 embryos	1.99 ± 0.11	1.98 ± 0.15	0.18
Blastocysts	1.59 ± 0.49	1.55 ± 0.50	0.51
OHSS rate (%)	15.48 (107/691)	11.75 (106/902)	0.03
Live birth rate (%)	95.55 (681/691)	98.78 (891/902)	0.69
GDM rate (%)	20.41 (141/691)	12.75 (115/902)	<0.001

BMI, body mass index; AMH, anti-Müllerian hormone; AFC, antral follicle count; FSH, follicle-stimulating hormone; LH, luteinizing hormone; E2, estradiol; PRL, prolactin; P, progesterone; T, testosterone; hCG, human chorionic gonadotropin; OHSS, ovarian hyperstimulation syndrome; GDM, gestational diabetes mellitus.

**Figure 1:**
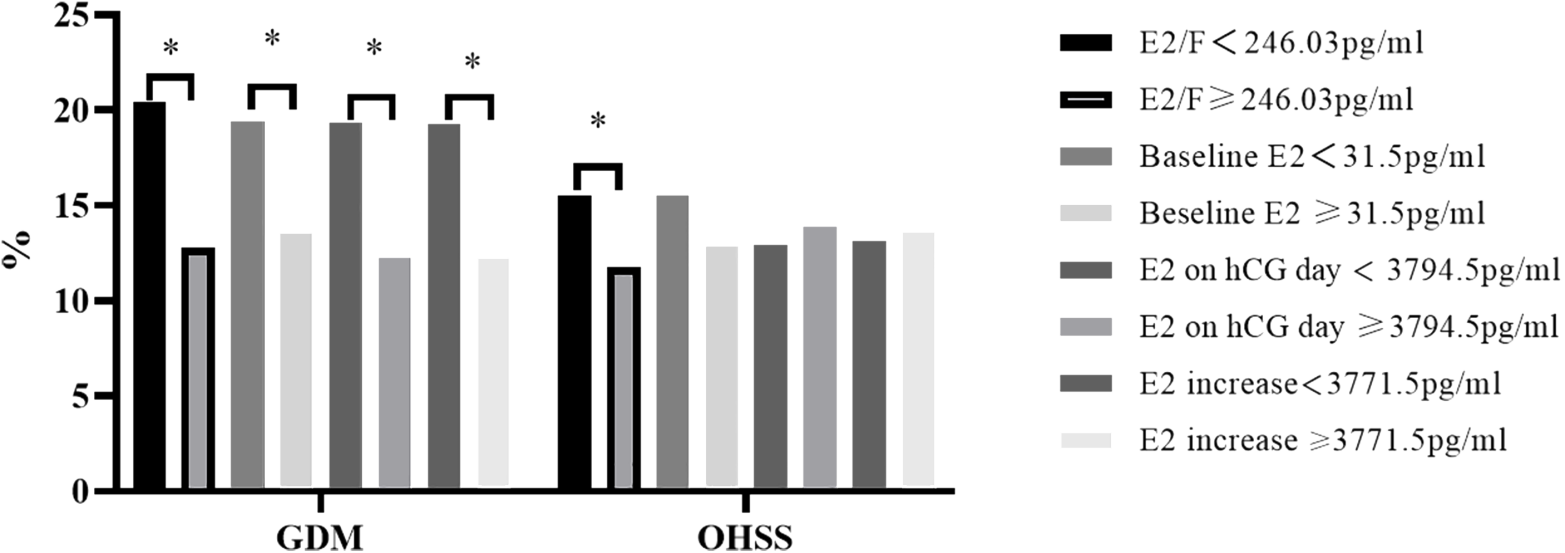
The GDM and OHSS rate in participants. OHSS, ovarian hyperstimulation syndrome. GDM, gestational diabetes mellitus. * represents P value < 0.05.

We also conducted a comparison of baseline metabolic factors potentially associated with GDM. The results reveal that blood pressure and fasting glucose levels (5.23 (4.99, 5.48) vs. 5.12 (4.90, 5.38), P<0.001) are lower in women with a higher E2/F ratio. Subsequently, significant differences in sex hormone levels on hCG day are observed, with higher LH (1.48 (1.15, 1.92) vs. 1.59 (1.25, 2.04), P<0.01), E2 (2760.00 (2122.00, 3568.00) vs. 4314.00 (3469.50, 5113.75), P<0.001), and progesterone (0.54 (0.38, 0.71) vs. 0.63 (0.47, 0.84), P<0.001) levels in women with a higher E2/F ratio. However, these women exhibit a lower follicle number on hCG day (14.00 (11.00, 18.00) vs. 13.00 (11.00, 16.00), P<0.001) (**Table 1**). Although no difference is observed in the live birth rate, the GDM rate (20.41% vs. 12.75%, P<0.001) and ovarian hyperstimulation syndrome (OHSS) rate (15.48% vs. 11.75%, P=0.03) is significantly lower in the higher E2/F ratio group (**Table 1**, **Figure 1**).

To account for confounding factors, our study employed regression analysis. Baseline characteristics were adjusted using univariate regression analysis, revealing that age, BMI, waist-to-hip ratio, baseline E2, baseline T, fasting glucose, and E2/F ratio are correlated with GDM (**Table 2**). All these significant parameters were subsequently included in a multivariate regression model, revealing that E2/F ratio plays a protective role in GDM (odds ratio (OR): 0.998, 95% confidence interval (CI): 0.997-1.000, P=0.04) (**Table 3A**, when E2/F ratio is treated as a continuous variable). Specifically, when the E2/F ratio is less than 246.03 pg/ml, it functions as a risk factor for GDM (OR: 1.626, 95% CI: 1.229-2.150, P<0.01) (**Table 3B**, when E2/F ratio is treated as a binary variable).

**Table 2:** Univariate regression analysis for baseline factors.

	B	Standard error	P value	OR	95% CI	
Age	0.129	0.021	<0.001	1.138	1.093	1.185
Menstrual cycle	-0.005	0.004	0.28	0.995	0.987	1.004
BMI	0.060	0.028	0.03	1.062	1.006	1.121
Waist to hip ratio	3.782	1.224	<0.01	43.887	3.985	483.325
Infertility duration	0.054	0.028	0.05	1.056	0.999	1.115
AMH	-0.027	0.015	0.07	0.973	0.945	1.002
AFC	-0.001	0.006	0.92	0.999	0.988	1.011
Basal FSH	-0.032	0.044	0.47	0.968	0.888	1.056
Basel LH	-0.039	0.024	0.10	0.962	0.918	1.008
Basal E2	-0.011	0.004	0.01	0.989	0.981	0.998
Basal PRL	0.006	0.004	0.12	1.006	0.999	1.014
Basal P	0.028	0.048	0.56	1.029	0.937	1.129
Basal T	0.038	0.018	0.04	1.039	1.002	1.077
Systolic blood pressure	0.004	0.007	0.55	1.004	0.991	1.017
Diastolic blood pressure	0.003	0.008	0.66	1.003	0.988	1.019
Fasting glucose	0.760	0.166	<0.001	2.139	1.545	2.962
E2/follicle ratio	-0.002	0.001	0.01	0.998	0.996	0.999
E2 on hCG day	0	0	<0.001	1.000	1.000	1.000
E2 fold change	-0.001	0.001	0.45	0.999	0.997	1.001
E2 increase	0	0	<0.001	1.000	1.000	1.000

OR, odd ratio; CI, confidential interval; BMI, body mass index; AMH, anti-Müllerian hormone; AFC, antral follicle count; FSH, follicle-stimulating hormone; LH, luteinizing hormone; E2, estradiol; PRL, prolactin; P, progesterone; T, testosterone.

**Table 3:** Multivariate regression analysis of all E2-related parameters.

A. Parameters as continuous variable.						
	B	Standard error	P value	OR	95% CI	
Basal E2	-0.011	0.004	0.01	0.989	0.981	0.998
E2/follicle ratio	-0.002	0.001	0.01	0.998	0.996	0.999
E2 on hCG day	0	0	<0.001	1.000	1.000	1.000
E2 increase	0	0	<0.001	1.000	1.000	1.000

B. Parameters as binary variable according to the cut-off value.						
	B	Standard error	P value	OR	95% CI	
Baseline E2< 31.5pg/ml	0.310	1.000	0.07	1.363	0.979	1.898
E2 on hCG day < 3794.5 pg/ml	0.413	1.000	0.01	1.511	1.133	2.016
E2 increase < 3771.50 pg/ml	0.420	1.000	<0.01	1.522	1.141	2.031
E2/F < 246.03 pg/ml	0.486	1.000	0.001	1.626	1.229	2.150

Confounding factors are: age, BMI, waist-to-hip ratio, fasting glucose, baseline T according to the baseline analysis.

OR, odd ratio; CI, confidential interval; E2, estradiol; hCG, human chorionic gonadotropin; F, follicle.

We also compared other E2-related parameters such as E2 on hCG day, baseline E2, E2 fold change (defined as E2 on hCG day divided by baseline E2), and E2 increase (defined as E2 on hCG day minus baseline E2) with GDM. The cut-off value defined for E2 on hCG day is 3794.50pg/ml (**Supplementary Figure S1B**). Women who acquired E2 on hCG day over 3794.50pg/ml had a lower GDM rate (12.26% (90/734) vs. 19.32% (166/859), P<0.001, **Figure 1**). Similarly, the baseline E2 over 31.50pg/ml (13.51% (122/903) vs. 19.42% (134/690), P<0.01) or the E2 increase over 3771.50pg/ml (12.24% (89/727) vs. 19.28% (167/866), P<0.001) also had a lower GDM rate than those who did not reach the level (**Figure 1**; **Supplementary Figures S1C, D**). However, the E2 fold change is not significant in predicting GDM incidence (**Supplementary Figure S1E**). Univariate and multivariate regression analysis also indicates that E2 on hCG day and E2 increase is negatively related to GDM (**Tables 2**, **3**). However, the OHSS rate is slightly higher in higher E2 in hCG day group and E2 increase group even though the P value is not significant (**Figure 1**).

## Discussion

### Main findings

In our study, we found that the E2/F ratio is significantly related to GDM, and we also defined a cut-off value of the E2/F ratio which is 246.03pg/ml. Women with an E2/F ratio over 246.03pg/ml have a decreased risk of GDM. We also observed the correlation between E2 on hCG day, E2 increase, and GDM, indicating the E2’s impact on GDM risk following IVF-induced pregnancy.

### Interpretation

Controlled ovarian hyperstimulation is an important step in the IVF procedure, aiming to retrieve multiple oocytes for IVF, resulting in more embryos for transfer and cryopreservation to increase pregnancy rates (24). Exogenous gonadotropins stimulate the growth and development of follicles, and as the follicles grow, the granulosa cells surrounding the follicles start to secrete E2. The E2 reaches peak level before ovulation which is on the day of hCG triggering in the IVF process. Serum E2 plays an important role in oocyte maturation and preparation of the uterus for implantation (25). The granulosa cells are the main source of E2, low E2/F indicates a poor growth of these granulosa cells which leads to a delayed hCG injection and subsequent oocyte retrieval and maturation (26). On the other hand, 17 β estradiol induces cytoplasmic maturation of germinal vesicle oocytes through an increase in intra-cytoplasmic calcium concentration, which affects further oocyte fertilization (27, 28). In women who received E2 supplementation after oocyte retrieval, significantly higher pregnancy and implantation rates were recorded, which also supports the favorable role of E2 in pregnancy during the IVF process (29).

The critical concentration of E2 affecting IVF outcomes such as GDM and ovarian hyperstimulation are still not well established. A system review and meta-analysis including 9 studies concludes that there is no high-quality evidence to support or deny the value of E2 determination on the day of hCG triggering final oocyte maturation for pregnancy achievement in IVF cycles due to the conflicting results in the analyzed studies (30). Another review also supports this conclusion (31). Current evidence reflects that serum E2 level is hard to determine the IVF outcomes since women reach different levels of serum E2 as the growing follicle number differs substantially due to differences in ovarian reserve in the individual women undergoing IVF treatment. Hence, Loumaye and colleagues suggested that the E2/F ratio could serve as a predictor of IVF success, and this concept was supported in subsequent studies (32).

Mittal S et al. found that E2/mature follicle (>14 mm) in 200-299.99pg/ml acquires the highest clinical pregnancy rate, and an increase of serum E2/F ratio is positively correlated with better oocytes and embryo quality (25). A low E2/F ratio seems to be associated with poorer oocyte and embryo quality as well as lower clinical pregnancy rate (33, 34) while a high E2/F ratio may be associated with increased oocyte number and quality (28). In these published studies concerning the E2/F ratio, only very few studies focused on the GDM risk. Thus, we designed this study to better understand the predictive value of the E2/F ratio on GDM risk.

In a study to investigate risk factors of GDM in the IVF process, the E2 level was found lower in the GDM group. Further results show the incidence of GDM was highest when the E2 level was less than 200 pg/mL per oocyte (35), which is similar to our study. However, in our study, the E2/F ratio also serves as a predictor for OHSS. E2 is found to protect against metabolic deterioration and GDM progression in obese mice models. An obvious improvement in impaired glucose tolerance was observed after the pregnancy in these mice. This is mediated by E2, which stimulates insulin secretion and improves hepatic glucose production, glucose uptake, and glycogen content in hepatocytes. This biological process is involved in activating the AKT pathway and intracellular cyclic adenosine monophosphate (cAMP) levels (36).

In our study, we also found that the E2 on hCG day over 3794.50pg/ml would play a protective role in GDM incidence. However, excessive E2 also contributes to OHSS, which is the most frequent complication of IVF treatment (37–39). Hence, studies suggested E2 threshold levels for reducing OHSS to be less than 3500pg/ml (40, 41), or even 3000pg/ml (42). Similarly, study also indicates that basal serum E2 showed a strong correlation with OHSS severity, with a cutoff value of 37.94 pg/ml (43). Taken together, higher E2 concentrations after ovarian hyperstimulation might increase the OHSS risk but do at the same time reduce the GDM risk on the other side. Our study suggests that the E2/F ratio could be a valuable tool to reliability recognize both risks with one test better than a simple analysis of E2. For these reasons, we believe the E2/F ratio is a better and safer predictor than other E2 parameters.

## Strengths and limitations

To the best of our knowledge, this is the first paper to compare several E2-related parameters with GDM incidence and OHSS rate, and we found the E2/F ratio is a better predictor. However, limitations also exist in our study. The predicting value only applies to women treated with the gonadotropin-releasing hormone (GnRH) agonist protocol, while it is unknown whether it is also suits for the GnRH-antagonist protocol or other protocols. Moreover, we did not exclude participant heterogeneity’s effect like other studies. For example, in women with polycystic ovarian syndrome, the follicle number is typically pretty high, which decreases the E2/F ratio since the E2 level is controlled to reduce the risk of OHSS during ovarian induction. This study was mainly done in Han Chinese women, replications in non-Chinese populations are needed.

## Conclusion

This study underscores the significance of the E2/F ratio as a predictive biomarker for GDM and OHSS in women undergoing fresh embryo transfer. By identifying an optimal E2/F ratio threshold of 246.03 pg/ml, we found that women exceeding this ratio experienced significantly lower rates of both GDM and OHSS. This relationship highlights the potential of the E2/F ratio to serve as a superior predictive marker compared to other estradiol-related parameters, such as baseline E2 levels and E2 levels on the day of hCG administration.

The findings from our research – if confirmed independent studies - may have implications for clinical practice, particularly in the management and treatment of patients undergoing IVF. By utilizing the E2/F ratio as a predictive tool, clinicians can better stratify patients based on their risk of developing GDM and OHSS. This stratification allows for more personalized treatment plans, potentially improving pregnancy outcomes and reducing the incidence of these complications. Early identification and intervention for high-risk patients can lead to more effective management strategies, including closer monitoring, dietary modifications, and timely medical interventions, thereby improving maternal and fetal health outcomes.

Further steps should involve validating these findings across diverse populations and different IVF protocols, including those using GnRH-antagonist protocols. Additionally, prospective studies could explore the underlying mechanisms linking estradiol levels and follicle numbers to GDM and OHSS risks. Understanding these mechanisms may provide new insights into the prevention and treatment of these conditions.

Moreover, integrating the E2/F ratio into routine clinical practice would require the development of standardized guidelines and training for healthcare professionals. Ensuring that the measurement of estradiol and follicle counts is consistent and accurate across different settings will be crucial for the widespread adoption of this predictive marker.

## Data Availability

The raw data supporting the conclusions of this article will be made available by the authors, without undue reservation.
