# Supplementary figures and images for: Estradiol-to-follicle ratio on human chorionic gonadotropin day is a novel predictor of gestational diabetes mellitus in women receiving fresh embryo transfer
Source: Front Endocrinol (Lausanne). 2024 Oct 11;15:1465069. doi: 10.3389/fendo.2024.1465069 (PMC11502309; doi:10.3389/fendo.2024.1465069)

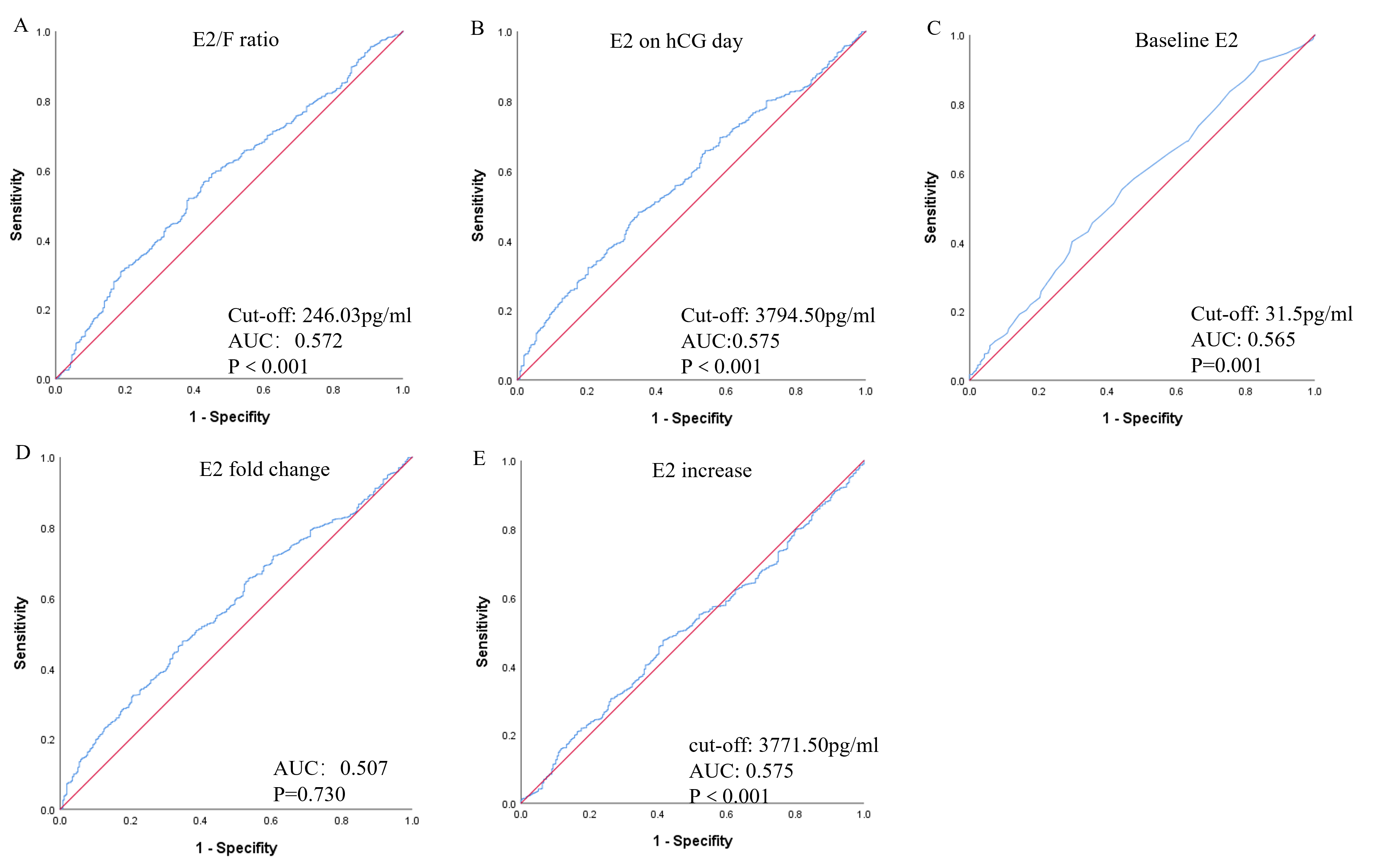

Supplement: Supplementary file 1 [file Image1.tif]
